# Assessing Effects of van der Waals Corrections on Elasticity of Mg_3_Bi_2−x_Sb_x_ in DFT Calculations

**DOI:** 10.3390/ma16196482

**Published:** 2023-09-29

**Authors:** Qing Peng, Xinjie Ma, Xiaoyu Yang, Shuai Zhao, Xiaoze Yuan, Xiaojia Chen

**Affiliations:** 1 School of Science, Harbin Institute of Technology, Shenzhen 518055, China; 2 The State Key Laboratory of Nonlinear Mechanics, Institute of Mechanics, Chinese Academy of Sciences, Beijing 100190, Chinayuanxz@imech.ac.cn (X.Y.); 3 Guangdong Aerospace Research Academy, Guangzhou 511458, China; 4 Beijing MaiGao MatCloud Technology Co., Ltd., Beijing 100190, China; 5 Computer Network Information Center, Chinese Academy of Sciences, Beijing 100190, China; 6 Department of Modern Mechanics, University of Science and Technology of China, Hefei 230026, China

**Keywords:** thermoelectric materials, PBE-D3, vdW-DFq, first-principles calculation, Mg_3_Bi_2−x_Sb_x_

## Abstract

As a promising room-temperature thermoelectric material, the elastic properties of Mg_3_Bi_2−x_Sb_x_ (0 ≤ x ≤ 2), in which the role of van der Waals interactions is still elusive, were herein investigated. We assessed the effects of two typical van der Waals corrections on the elasticity of Mg_3_Bi_2−x_Sb_x_ nanocomposites using first-principles calculations within the frame of density functional theory. The two van der Waals correction methods, PBE-D3 and vdW-DFq, were examined and compared to PBE functionals without van der Waals correction. Interestingly, our findings reveal that the lattice constant of the system shrinks by approximately 1% when the PBE-D3 interaction is included. This leads to significant changes in certain mechanical properties. We conducted a comprehensive assessment of the elastic performance of Mg_3_Bi_2−x_Sb_x_, including Young’s modulus, Poisson’s ratio, bulk modulus, etc., for different concentration of Sb in a 40-atom simulation box. The presence or absence of van der Waals corrections does not change the trend of elasticity with respect to the concentration of Sb; instead, it affects the absolute values. Our investigation not only clarifies the influence of van der Waals correction methods on the elasticity of Mg_3_Bi_2−x_Sb_x_, but could also help inform the material design of room-temperature thermoelectric devices, as well as the development of vdW corrections in DFT calculations.

## 1. Introduction

The thermoelectric effect is a phenomenon in which a material generates an electric current when exposed to a temperature gradient. Materials with exceptional thermoelectric properties have significant applications in energy conversion processes, such as refrigeration and waste heat harvesting [1,2]. In recent years, there has been a growing interest in investigating the thermoelectric properties of Mg_3_Bi_2−x_Sb_x_ [3,4,5,6,7,8,9,10,11], a promising room-temperature thermoelectric material. Notably, Mg_3_Bi_2−x_Sb_x_ exhibits a remarkable thermoelectric figure of merit, reaching approximately 1.5 when x = 0.5 [12]. This places it in a competitive position when compared to more traditional thermoelectric materials like Bi_2_Te_3_ [13] and Ag_2_Se [14], offering advantages such as cost-effectiveness. Researchers have delved into various aspects of Mg_3_Bi_2−x_Sb_x_ over the past few years. These investigations have covered various areas, including band topology [15,16,17,18], phonon dynamics [19,20,21,22,23], and the topological thermoelectric properties of nodal-line semimetals [22,23,24,25,26,27]. Notable contributions, such as the work of Kanno [28], have shed light on specific characteristics of Mg_3_Bi_2−x_Sb_x_. For instance, it has been demonstrated that this material exhibits higher charge carrier mobility and lower thermal conductivity, which can be attributed to its disordered structure.

As a prospective room-temperature high-performance thermoelectric material, it is crucial to investigate its mechanical properties. Despite prior computational investigations into the elastic characteristics of Mg_3_Bi_2−x_Sb_x_ using first principles, as seen in works like those by Peng et al. [29], the consideration of van der Waals (vdW) correction is noticeably absent although desirable. The interactions due to the fluctuation of nonlocal charge densities are hard to model but crucial in first-principles calculations. To improve the accuracy of our calculations and understand the impact of vdW correction within the system, we conducted a comprehensive comparative study employing both the vdW-DFq [30] and PBE-D3 [31] methods with reference to the impact of non-vdW correction (and thus, PBE). Our analysis revealed intriguing findings: the inclusion of vdW interactions resulted in a reduction in lattice constants, particularly noticeable using the PBE-D3 method, which displayed a more significant decrease of approximately 1%. Importantly, the PBE-D3 calculations demonstrated a closer alignment with experimental data, thereby augmenting their persuasiveness. Our investigation encompassed a thorough exploration of 40-atom systems with varying Sb content. Remarkably, except for the specific point at x = 0.375, neither the PBE-D3 nor the vdW-DFq methods altered the trend of elastic properties in Mg_3_Bi_2−x_Sb_x_ as the Sb content varied. The changes were solely confined to absolute values, usually within a range of about 10%. This comprehensive analysis not only enhances our understanding of the mechanical behavior of Mg_3_Bi_2−x_Sb_x_, but also underscores the nuanced yet pivotal role of vdW correction.

## 2. Materials and Methods

**Figure 1:**
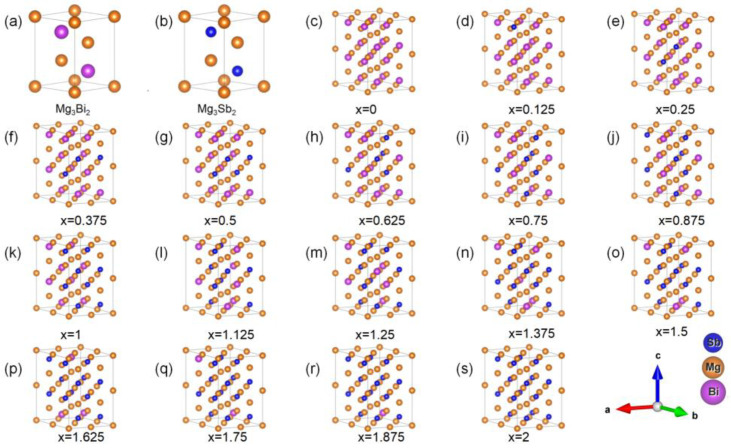
The atomistic structures of Mg_3_Bi_2−x_Sb_x_: the 5-atom unit cell of (**a**) Mg_3_Bi_2_ and (**b**) Mg_3_Sb_2_, and (**c**–**s**) the 40-atom cells of Mg_3_Bi_2−x_Sb_x_ for x ranging from 0 to 2 with increments of 0.125.

Both Mg_3_Bi_2_ and Mg_3_Sb_2_ belong to the trigonal crystal system, with a symmetry that belongs to the space group P$\overset{-}{3}$m1. We have plotted all the structures in Figure 1. The primitive unit cell consists of five atoms, as shown in Figure 1a,b for Mg_3_Bi_2_ and Mg_3_Sb_2_, respectively. In the case of Mg_3_Bi_2−x_Sb_x_, the objective is to maintain a constant number of Mg atoms while gradually substituting Bi atoms with Sb atoms. To cover a wide range of concentrations, we expanded the original unit cell to a 2 × 2 × 2 supercell, resulting in a total of 40 atoms. Among these atoms, there are 16 atoms that are either Bi or Sb, with concentrations ranging from 0 to 16 Sb atoms, as shown in Figure 1c–s. The initial structures of these 17 configurations were obtained from LAsou [32,33]. LAsou is an active learning approach that overcomes the exponential-wall problem for the effective structure prediction of chemical-disordered materials. The lowest-energy configuration was chosen as the representative structure for that particular concentration. For the calculation of elastic constants, each representative structure was further optimized to a higher precision. We applied both positive and negative strains to the six independent degrees of freedom in the system, resulting in a total of 12 different configurations. Through first-principles calculations, we determined the internal pressures for these 12 configurations, enabling us to obtain the elastic constants for the entire system.

To obtain the energies and stresses of configurations, we utilized the Vienna Ab initio Simulation Package (VASP) [34,35,36,37], a piece of software based on first-principles density functional theory. The Perdew–Burke–Ernzerhof (PBE) [37] exchange-correlation functional was chosen for the calculations. For the 40-atom system calculations, we utilized the Methfessel–Paxton [38] method to generate k-points centered at the gamma point, with a grid setting of 4 × 4 × 2. The energy cutoff for the plane wave basis used in the calculations was 450 eV. During structural optimization, a force convergence criterion of 10^−3^ eV/Å was used, while the electronic convergence criterion was 10^−6^ eV. We adopted Gaussian smearing and the smearing width was 0.02 eV. We used VASPKIT [39] to process the obtained elastic constants tensor, allowing us to extract Young’s moduli and Poisson’s ratios for different directions [40]. Given the significant number of configurations, we integrated the calculation workflow onto the MatCloud [41] platform for improved efficiency. This cloud-based approach allowed us to efficiently distribute multiple jobs to high-performance computing clusters, optimizing resource utilization for extensive system calculations.

For vdW corrections, two distinct approaches were utilized: PBE-D3 and vdW-DFq. The PBE-D3 method, which was developed by Grimme et al. [30], is designed to accurately model the vdW dispersion energy-correction term. The incorporation of empirical parameters in density functional theory has been proposed as a means to address its limitations. This approach has shown promising results, especially in the case of systems that involve heavy elements. On the contrary, vdW-DFq correction, as proposed by Peng et al. [31], presents a novel approach for precise density and geometry calculations, with a specific focus on semihard materials. Both of these methods have been effectively integrated into the VASP-5.4.4 software. By manipulating various parameters, we successfully performed a range of calculations using these methodologies. PBE-D3 addresses the limitations of density functional theory by integrating empirical parameters, whereas vdW-DFq prioritizes the precise computation of the density and geometric characteristics of semihard materials.

## 3. Results

**Figure 2:**
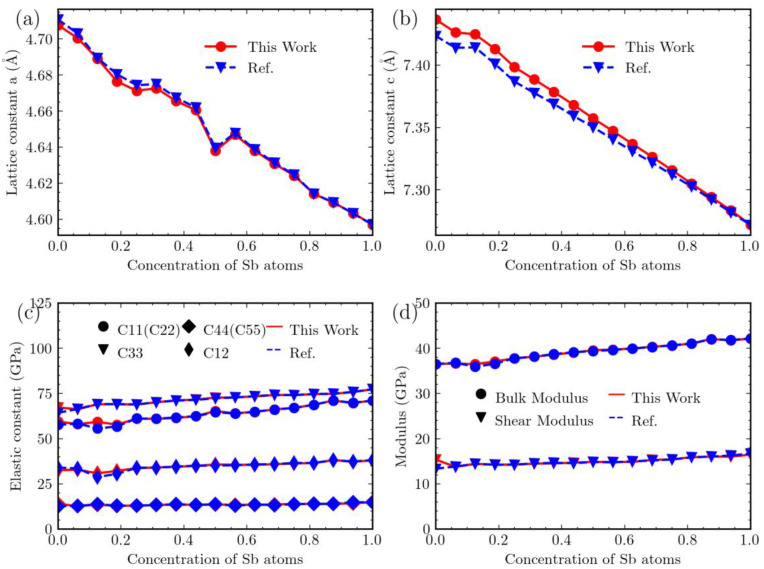
Lattice constants *a* (**a**) and *c* (**b**), elastic constants (**c**), and elastic modulus (**d**) of Mg_3_Bi_2−x_Sb_x_ as a function of the concentration of Sb atoms, compared with ref. [29]. The red lines denote values from this work and the blue ones represent values from ref. [29].

Prior to examining the vdW corrections, we conducted a reference study [29] to compare the effects of precision enhancement on various system parameters without considering vdW correction. This comparison encompasses lattice constants “*a*” (in Å) and “*c*” (in Å), elastic constants C_11_, C_33_, C_44_, and C_12_, as well as bulk and shear moduli (in GPa). The results of our precision enhancement are shown in red, while the results from previous work are shown in blue, in Figure 2.

Upon comparison, it becomes evident that the impact of precision enhancement on lattice constant “*a*” is not particularly significant. However, for lattice constant “*c*”, especially at lower values of Sb content, the impact is relatively more significant. In contrast, for higher concentrations of Sb, the precision used in previous calculations appears to be sufficient. Regarding elastic constants and moduli, the impact of enhancing precision is usually negligible, except for the Mg_3_Bi_2_ system, which exhibits more noticeable variations. These comparisons suggest that when performing calculations on Mg_3_Bi_2−x_Sb_x_, it may be possible to decrease precision to some extent in systems with higher Sb content in order to improve computational efficiency, while still maintaining acceptable accuracy. Conversely, for systems with lower Sb content, it is advisable to increase precision.

The comparison with previous work [29] indicates that precision adjustments in Mg_3_Bi_2−x_Sb_x_ calculations can be customized based on the Sb concentration in the system. It is recommended to use higher-precision for systems with lower Sb content to ensure accuracy, while systems with higher Sb content can benefit from reduced precision to improve computational efficiency. Additionally, this study adds credibility to our current efforts involving the inclusion of vdW corrections.

**Figure 3:**
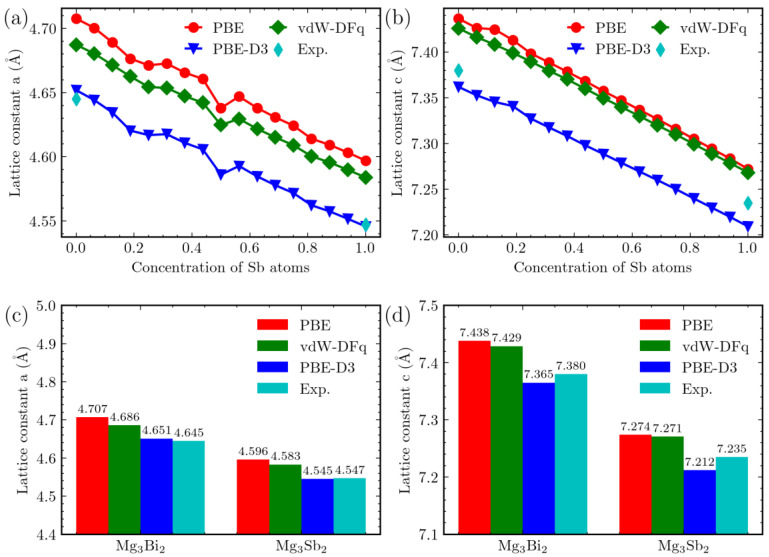
Lattice constants of Mg_3_Bi_2−x_Sb_x_. The four methods are color-coded: red (PBE), green (vdW-DFq), blue (PBE-D3), and cyan (experimental data [42]). (**a**) The trend of lattice constant “*a*” with respect to Sb content. (**b**) Lattice constant “*c*” as a function of Sb content. (**c**) Comparison of lattice constant “*a*” among different Mg_3_Bi_2_ and Mg_3_Sb_2_ structures using various methods. (**d**) Comparison of lattice constant “*c*” among different Mg_3_Bi_2_ and Mg_3_Sb_2_ structures using various methods.

All the calculated configurations of Mg_3_Bi_2−x_Sb_x_ belong to the trigonal crystal system. Using first-principles calculations, we obtained the optimal structures for each configuration. For the trigonal crystal system, our focus was on lattice constants “*a*” and “*c*”. We plotted the lattice constants that vary with different Sb atom content in Figure 3. It is clear from Figure 3 that, regardless of the calculation method used, the lattice constants decrease as the Sb atom content increases. Considering the impact of different functionals, it can be observed that the influence of vdW-DFq on the lattice constants is minimal. For lattice constant “*a*”, there is a decrease of approximately 0.4%. The effect on lattice constant “*c*” is negligible. However, PBE-D3 correction has a relatively more noticeable impact. Taking the Mg_3_Bi_2_ system as an example, lattice constant “*a*” decreases by 1.3%, while lattice constant “*c*” is affected by 1.1%.

We conducted a comparison between the experimental lattice constants of Mg_3_Bi_2_ and Mg_3_Sb_2_, as illustrated in Figure 3 c,d, respectively. The figure clearly demonstrates a close alignment between the experimental lattice constants and those obtained through the application of the PBE-D3 method. Therefore, this comparison provides robust evidence in favor of employing PBE-D3 correction in calculations related to Mg_3_Bi_2−x_Sb_x_.

**Figure 4:**
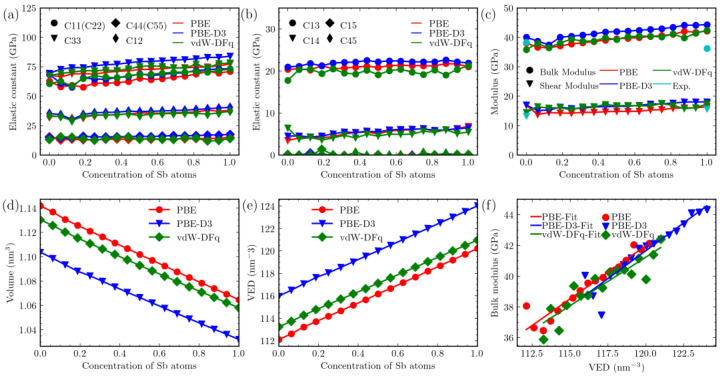
Comparison of elastic constants from different calculation methods: red (PBE), green (vdW-DFq), blue (PBE-D3), cyan (Exp.). (**a**,**b**) Elastic constants, (**c**) bulk modulus and shear modulus, (**d**) volume, (**e**) valence electron density (VED), (**f**) bulk modulus with changing average VED.

Upon subjecting the system to deformation along its six independent degrees of freedom, we obtained a 6 × 6 elastic constant tensor. For the trigonal crystal system, the elastic tensor possesses eight independent components, namely, C_11_, C_33_, C_44_, C_12_, C_13_, C_14_, C_15_, and C_45_. We have illustrated the variations in Figure 4a,b, depicting the changes in Sb content. Regardless of the adoption of vdW functionals, the values of these components exhibit a linear increase as x changes, except for the points at Sb concentrations of 0.1875 and 0.25 (corresponding to x = 0.375 and x = 0.5). Specifically, for the C_44_ component, the PBE-D3 calculations generally yield higher results than those without vdW corrections, showing an increase of approximately 16%, except for the pure Mg_3_Bi_2_ state. Comparatively, the results of vdW-DFq exhibit some variation, with values oscillating and differing by approximately 8% from the PBE calculations.

For the C_12_ component, it is noticeable that the results from all three methods are quite similar. Comparing the results obtained from vdW-DFq with those obtained using only PBE without considering vdW corrections, there are some variations in the values for different structures within Mg_3_Bi_2−x_Sb_x_, with an average change of approximately 2.5%. For PBE-D3, the results for all structures, except for the one at an Sb concentration of 0.1875, are generally larger than those obtained with PBE. The structure at x = 0.375 is about 2.3% smaller than PBE. Overall, there is a numerical difference of about 5.5%.

Concerning the C_33_ component, once again, the results from vdW-DFq and PBE are relatively close, with differences of approximately 2.4%. However, the results obtained with PBE-D3 are, on average, about 8.8% larger than those obtained with PBE. As for the C_44_ component, all methods show a decrease at an Sb concentration of 0.1875, while at other points, they increase with increasing Sb content. In the calculation of this component, the influence of vdW is more significant. Compared to PBE calculations, the results from vdW-DFq are, on average, 8.3% higher, while the results from PBE-D3 are, on average, 16.8% higher. As for the components in Figure 4b, C_15_ and C_45_ are both close to 0 overall, while both C_13_ and C_14_ show an increase with Sb content. The results obtained from various vdW methods differ from those of the PBE method by approximately 6%.

Typically, the bulk modulus is used to describe a material’s resistance to compression and expansion in volume, while the shear modulus characterizes its resistance to shearing forces. In the context of crystalline systems, both the bulk and shear moduli exhibit varying degrees of anisotropy. To facilitate the comparative analysis of the variation in bulk and shear moduli for different Sb contents, we adopted the Voigt-Reuss-Hill approximation [43,44,45] to describe the changes in these moduli. We utilized the following formulas to calculate the bulk modulus and shear modulus:

(1) $$B=\frac{1}{2}\left(B_{V}+B_{R}\right)\;G=\frac{1}{2}\left(G_{V}+G_{R}\right)$$

where B_V_, B_R_, G_V_, and G_R_ can be obtained from the following expressions based on the elastic constant and its compliance S, which is often used to describe the compliance of materials and can be calculated by taking the inverse of the elastic tensor:

(2) $$\begin{array}{l} B_{V}=\frac{1}{9}\left(C_{11}+C_{22}+C_{33}\right)+\frac{2}{9}\left(C_{23}+C_{13}+C_{12}\right)\\ B_{R}=\frac{1}{3\left(S_{11}+S_{22}+S_{33}\right)+6\left(S_{23}+S_{13}+S_{12}\right)} \end{array}$$

(3) $$\begin{array}{l} G_{V}=\frac{1}{5}\left[\left(C_{11}+C_{22}+C_{33}\right)-\left(C_{23}+C_{13}+C_{12}\right)\right]+\frac{3}{5}(C_{44}+C_{55}+C_{66})\\ G_{R}=\frac{1}{12\left[\left(S_{11}+S_{22}+S_{33}\right)-\left(S_{23}+S_{13}+S_{12}\right)\right]+9\left(\left(S_{44}+S_{55}+S_{66}\right)\right)} \end{array}$$

This relationship is depicted in Figure 4c. It is evident that with increasing Sb content, except for a few points, both the shear and bulk moduli show an increase. Regarding the influence of vdW corrections on the bulk modulus, the results obtained from the PBE-D3 calculations are generally higher compared to those from vdW-DFq and PBE. Taking Mg_3_Sb_2_ as an example, the bulk moduli calculated using the three methods are 44.34 GPa (PBE-D3), 42.41 GPa (vdW-DFq), and 42.14 GPa (PBE), respectively. The PBE-D3 results are approximately 5.2% higher than the PBE results. Overall, the bulk moduli obtained using PBE-D3 are approximately 5.5% higher than those obtained without vdW corrections. In comparison, the impact of vdW-DFq is relatively smaller, with numerical differences of approximately 2%.

Concerning the shear modulus, both the PBE-D3 and vdW-DFq results are higher by about 10% compared to the PBE results. However, there are some minor differences in the details. For instance, in the case of PBE-D3, the result at the point where the Sb concentration is 0.1875 is similar to those obtained without vdW corrections. On the other hand, for vdW-DFq, the points at Sb concentrations of 0.9375 and 0.875 (corresponding x = 1.875 and x = 1.75) closely align with the PBE results. This alignment is understandable because the corresponding moduli are derived from the elements of the elastic constants’ matrix, and their trends also align with the trends of the elastic constants. For comparison, we have indicated the experimental [46] values for the bulk and shear moduli of Mg_3_Bi_2_ and Mg_3_Sb_2_ as cyan dashed points in Figure 4c. It can be observed that, except for the bulk modulus of Mg_3_Sb_2_, the calculated moduli closely match the experimental values.

We have simultaneously illustrated the trends in volume, bulk modulus, and average valence electron density (VED). VED is defined as the number of valence electrons in a unit volume. These trends are compared for the three methods with respect to the concentration of Sb atoms, as shown in Figure 4. Since the numbers of valence electrons for the elements Mg, Bi, and Sb are 2, 5, and 5, respectively, all 40-atom cell structures of Mg_3_Bi_2−x_Sb_x_ have the same number of total valence electrons, which is 128. Due to the disparities in lattice constants obtained from different calculation methods, these differences are further magnified at the volume level. The lattice volume decreases as the content of Sb increases. Additionally, when employing the PBE-D3 method for vdW simulations, the volume is approximately 5% smaller than when not using vdW corrections. It is worth noting that VED is different from valence electron concentration (VEC), which is defined as the number of valence electrons in a formula unit [47].

According to the findings of a previous study [29], in the Mg_3_Bi_2−x_Sb_x_ system, except for the special point at an Sb concentration of 0.25, the bulk modulus demonstrates a linear relationship with the VED. This conclusion is supported by the observations in Figure 4f. However, when improving computational precision, the linear relationship becomes less noticeable when using the vdW-DFq results. In contrast, the results from PBE-D3 still exhibit a linearly increasing trend, although with some deviations at several points for x < 0.5. Such a trend can be understood as follows: a higher valence electron density implies stronger atomic interactions, and consequently, a higher bulk modulus. This ascending trend indicates that we can intentionally adjust the relevant elastic properties of the material by consciously manipulating VED, such as through doping with other elements like Co and Te [28,48].

Furthermore, we employed Jiang’s model [49] to investigate the Vickers hardness of the Mg_3_Bi_2−x_Sb_x_ system. This empirical model establishes a linear relationship between the Vickers hardness and Young’s modulus. Utilizing the previously calculated bulk and shear moduli, we can derive the Young’s modulus (E) using the following formula. Subsequently, we can deduce the Vickers hardness (V):

(4) $$\begin{array}{c} E=\frac{9GB}{3B+G}\\ V=0.0608E \end{array}$$

**Figure 5:**
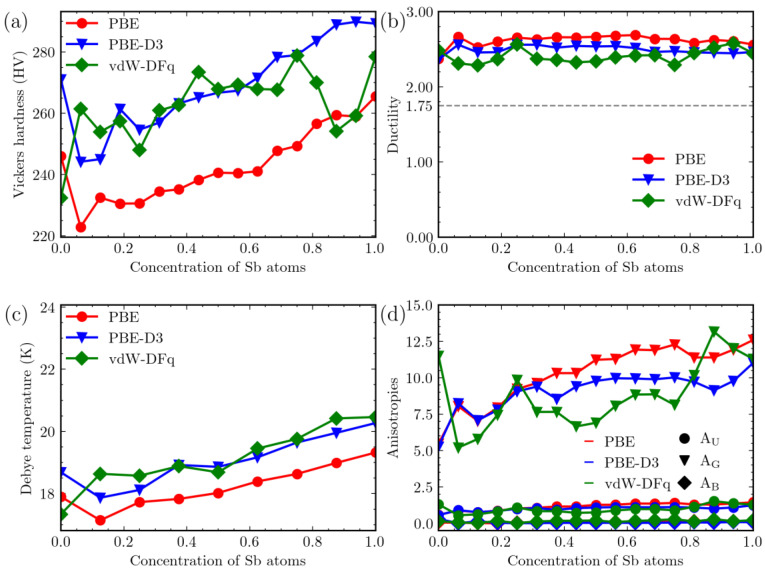
Comparison of (**a**) Vickers hardness, (**b**) ductility, (**c**) Debye temperature and (**d**) anisotropies from the three methods. Red (PBE), green (vdW-DFq), blue (PBE-D3).

We calculated the Young’s Modulus and the corresponding Vickers hardness (in HV) for various Sb concentrations in Mg_3_Bi_2−x_Sb_x_ and graphed the hardness values in Figure 5a. For the Mg_3_Sb_2_ system, we obtained Young’s Modulus values of 43.69 GPa, 47.58 GPa, and 45.82 GPa, using the PBE, PBE-D3, and vdW-DFq methods, respectively. These values are in close agreement with the experimental Young’s Modulus of approximately 41 GPa for Mg_3_Sb_2_, indicating good consistency between our calculations and experimental data [46].

As the Sb content increases, the hardness also shows an upward trend. The three calculation methods exhibit slight discrepancies in detail. The overall hardness calculated using the PBE-D3 and vdW-DFq methods is higher than that obtained without considering vdW corrections. However, at the point of Mg_3_Bi_2_, the results obtained using vdW-DFq are lower than those from PBE, by approximately 5.5%. At Sb concentrations of 0.9375 and 0.875, the vdW-DFq result closely resembles that without vdW, while the PBE-D3 result is significantly higher.

Similarly, the Pugh ratio [50] was employed to evaluate the ductility of the system. The Pugh ratio is derived using a specific formula (B/G), where the symbols B and G in the equation represent the bulk modulus and shear modulus, respectively. The outcomes of this comparison are graphically represented in Figure 5b. Regardless of the computational approach employed, the outcomes exhibit a range between 2 and 3. According to empirical observations, materials that have a Pugh ratio greater than 1.75 are regarded as exhibiting toughness. Consequently, all concentrations within the system under study can be considered as demonstrating resilience. In contrast, the widely recognized thermoelectric material Bi_2_Te_3_ exhibits a Pugh ratio of 1.62, suggesting that the Mg_3_Bi_2−x_Sb_x_ system possesses relatively lower brittleness in comparison.

The Debye temperature [51] is a parameter that represents the highest-frequency lattice vibrations and is commonly employed as an indicator of the strength of interatomic bonding. It can be determined by employing the following formula:

(5) $$\Theta =\frac{h}{k_{B}}{\left(\frac{3q}{4\pi }\frac{N\rho }{M}\right)}^{\frac{1}{3}}v_{m}$$

where h, k_B_, and N are the Planck constant, Boltzmann constant, and Avogadro constant, respectively; *ρ* is the mass density; M is the molecular weight of all the atoms in the supercell; and q is the number of atoms in the unit cell. The average sound velocity (v_m_) is defined as a combination of the shear sound velocity (v_s_) and the longitudinal sound velocity (v_l_):

(6) $$v_{m}={\left[\frac{1}{3}\left(\frac{2}{v_{s}^{3}}+\frac{1}{v_{l}^{3}}\right)\right]}^{-\frac{1}{3}}$$

where $v_{s}=\sqrt{G/\rho }$ and $v_{l}=\sqrt{(B+\frac{3}{4}G)/\rho }$, respectively.

The results of the Debye temperature calculations are presented in Figure 5c. Here, it is apparent that the inclusion of PBE-D3 and vdW-DFq increases the Debye temperature compared to cases where vdW is not considered, and the value is roughly 7% higher when PBE-D3 is used. As the content of Sb increases, there is a corresponding increase in the Debye temperature, indicating strengthening of the interatomic bonding within the system. The results obtained using the vdW-DFq method generally align with the trend observed when vdW correction is not considered.

We describe the anisotropy of the system through three indices, A_U_, A_G_, and A_B_, which are obtained using the following formula [52,53]:

(7) $$\begin{array}{l} A_{U}=\frac{5G_{V}}{G_{R}}+\frac{B_{V}}{B_{R}}-6\\ A_{G}=\frac{G_{V}-G_{R}}{G_{V}+G_{R}}\times 100\\ A_{B}=\frac{B_{V}-B_{R}}{B_{V}+B_{R}}\times 100 \end{array}$$

We have presented the results in Figure 5d, where A_B_ is observed to be close to 0. This suggests that the system exhibits relatively consistent properties in terms of bulk modulus across different crystallographic directions. This observation is further confirmed by the 3D plot of the bulk modulus. The maximum and minimum values of the bulk modulus in different directions for all systems are within 7 GPa. In contrast, when compared to Bi_2_Te_3_, which has an A_B_ value of 1.58, indicating strong anisotropy as it exceeds 1, Mg_3_Bi_2−x_Sb_x_ exhibits significantly lower anisotropy. In the case of the shear modulus, the systems at various concentrations exhibit noticeable anisotropy due to the relatively large values of A_G_. This anisotropy increases with higher Sb content. From the perspective of the A_U_ parameter, Mg_3_Bi_2−x_Sb_x_ exhibits significant directionality when compared to other alloys such as Zr_5_Sn_3_X (X = B, Nb, and Sn), where their A_U_ values are all within 0.5. Except for Mg_3_Bi_2_, all other Sb concentrations have A_U_ values above 0.7, and these values increase with higher Sb content. When considering the A_U_ parameter, the use of vdW introduces some bias in the results, but it does not affect the overall trend with increasing Sb content.

**Figure 6:**
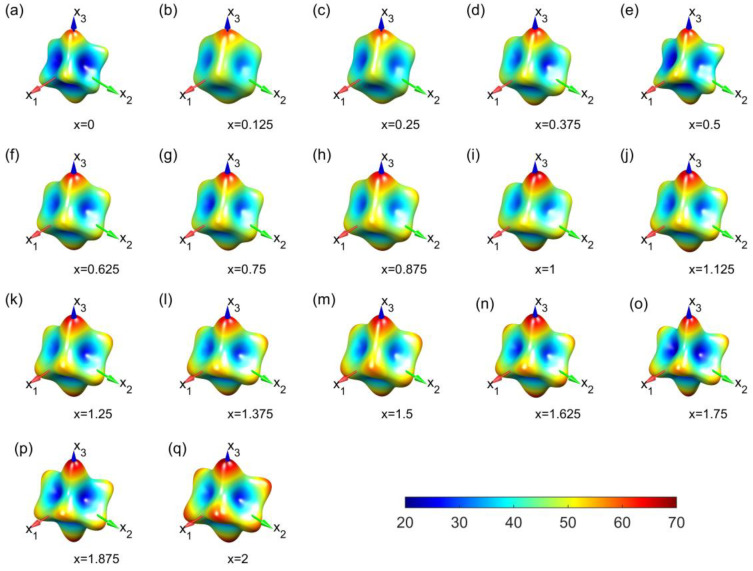
Three-dimensional Young’s modulus distribution of Mg_3_Bi_2−x_Sb_x_ (0 ≤ x ≤ 2) at various x of Sb contents (**a**–**q**) from the vdW-DFq calculations. Direction-dependent Young’s modulus for every potency of Sb at an interval of 0.125, ranging from 0 to 2. The color bar shows the rage of Young’s modulus with unit of GPa.

To enhance our understanding of the mechanical anisotropy of Mg_3_Bi_2−x_Sb_x_, we employed VASPKIT to extract Young’s moduli for different crystallographic orientations. We then visualized these data as 3D surface plots, where the distance from the origin to each point on the surface represents the Young’s modulus in the corresponding direction. Furthermore, to visualize the magnitude of Young’s modulus more conveniently, we used different colors on the surface to represent different values of Young’s modulus. In Figure 6, we present the Young’s modulus for different Sb concentrations, obtained via the vdW-DFq calculation method.

It is evident that, for all concentrations, the maximum Young’s modulus is obtained along the x_3_ direction, which corresponds to the lattice vector “***c***” within the crystal cell. All surfaces for the systems take the form of a hexahedron (a six-faced figure), with the degree of concavity varying at different concentrations, reflecting differences in anisotropy. Within the x_1_–x_2_ plane, the edges of these hexahedra are visible. In these materials, the Young’s modulus is relatively high. The manifestation of this characteristic is primarily due to the occupation of Mg^2+^ ions at the symmetric positions 3 m and −3 m within the unit cell. As the Sb content increases, it can be observed that the surface expands significantly. This also implies that the Young’s modulus is increasing in all directions. As mentioned earlier, this is related to the enhanced atomic interactions resulting from the addition of Sb atoms.

**Figure 7:**
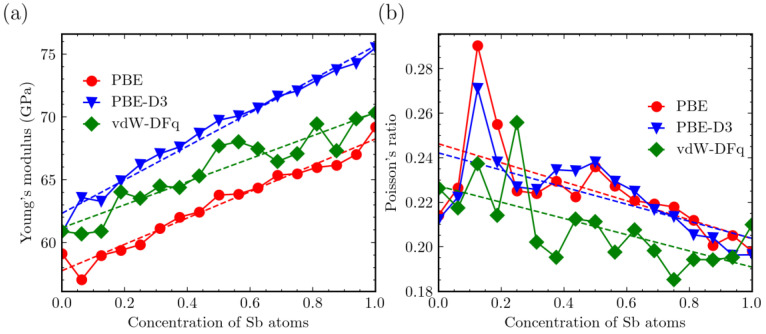
Young’s modulus and Poisson’s ratio from the three methods: red (PBE), green (vdW-DFq), blue (PBE-D3). (**a**) Trends in Young’s Modulus in x_3_ direction as function of Sb atom concentration. (**b**) Variation in Poisson’s ratio in the x_3_ direction with changing Sb concentration.

We examined the Young’s modulus in the x_3_ direction using the three calculation methods. The results are displayed in Figure 7a. In all other cases, the Young’s modulus in the x_3_ direction ranges from 55 GPa to 75 GPa. When vdW corrections are taken into account, the Young’s modulus in the x_3_ direction shows a linear increase with increasing Sb content. However, for the vdW-DFq calculations, this linear trend deviates somewhat. Nevertheless, the values obtained with vdW-DFq are relatively close to those obtained without vdW corrections.

Considering the influence of vdW corrections, the Young’s modulus was generally higher than when these vdW corrections were not considered. This aligns with expectations because both an increase in Sb content and the inclusion of vdW corrections lead to a reduction in lattice constants. This reduction results in closer atomic distances and stronger interatomic interactions, which consequently yield higher Young’s moduli.

When a material is subjected to deformation in one direction, it also undergoes strain in the transverse directions in general. This phenomenon is known as the Poisson effect. Poisson’s ratio is used to measure this effect and is defined as the ratio of transverse strain to longitudinal strain. Poisson’s ratio reflects a material’s hardness to some extent. Similar to Young’s modulus, Poisson’s ratio of Mg_3_Bi_2−x_Sb_x_ also exhibits significant anisotropy.

**Figure 8:**
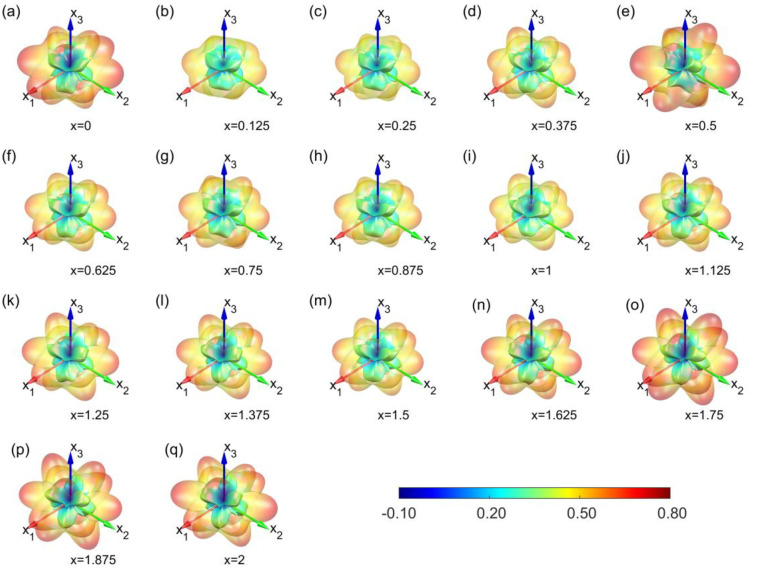
Poisson’s ratio distribution of Mg_3_Bi_2−x_Sb_x_ (0 ≤ x ≤ 2) at various Sb contents. (**a**–**q**) Direction-dependent Poisson’s ratio at different Sb contents of x, ranging from 0 to 2, calculated via vdW-DFq. Outer surface denotes the maximum Poisson’s ratio and inner surface is the contrary.

To better visualize this anisotropy, we created 3D plots of the Poisson’s ratio for various Sb concentrations, as shown in Figure 8. These results are based on calculations with vdW-DFq for the vdW corrections. Unlike Young’s modulus, Poisson’s ratio displays irregularities, with values ranging from −0.1 to 0.8 in different directions. In certain directions, Poisson’s ratio approaches zero or even becomes negative. This indicates that stretching in these directions does not result in transverse contraction, which is a phenomenon that is less commonly observed in traditional alloys.

Similarly, to compare the influence of vdW corrections, we examined Poisson’s ratio in the x_3_ direction, as depicted in Figure 7b. With few exceptions, the Poisson’s ratio in the x_3_ direction of Mg_3_Bi_2−x_Sb_x_ decreases linearly with increasing Sb content. With the inclusion of vdW effects, for the PBE-D3 method, the Poisson’s ratio results are relatively close to those without vdW. In contrast, for the vdW-DFq method, the results are slightly lower than those without vdW, but its linear trend of decreasing with increasing Sb content is more pronounced.

## 4. Conclusions

A systematic investigation was conducted into the mechanical properties of the room-temperature thermoelectric material Mg_3_Bi_2−x_Sb_x_ (0 ≤ x ≤ 2) through the utilization of first-principles calculations within the frame of density functional theory. By conducting a comparative analysis of the impacts of distinct vdW corrections, we have provided a comprehensive understanding of the computational performance of these three methods and their outcomes. The influence of precision is contingent upon the concentration of Sb atoms. Higher-precision calculations have a negligible impact on the final optimized lattice constants, and this impact diminishes as the concentration of Sb increases. Additionally, in the context of elastic constants and elasticity, it was found that higher precision has minimal impact, except for the Mg_3_Bi_2_ system.

We conducted a comprehensive assessment of the influence of vdW corrections on the elasticity of the Mg_3_Bi_2−x_Sb_x_ (0 ≤ x ≤ 2) nanocomposites. We employed both the PBE-D3 and vdW-DFq methodologies for nonlocal vdW corrections. The PBE-D3 method has a discernible effect of approximately 1% on lattice constants. In terms of other properties, such as elastic constant and bulk modulus, the influence of this method is generally within a range of 10%, except for a few outliers. The vdW-DFq method has a limited influence. Furthermore, the inclusion of vdW interactions does not significantly alter the patterns observed in various properties as the concentration of Sb vary, except for a few anomalous data points. In the case of lattice constants, the results obtained using the PBE-D3 method are closer to the experimental values.

Our results suggest the necessity of employing the PBE-D3 method to consider vdW corrections for more accurate prediction of the properties of Mg_3_Bi_2−x_Sb_x_ by means of density functional theory calculations. This assessment might be helpful in the further development of vdW corrections and the material design of room-temperature thermoelectric materials.

## Data Availability

Not applicable.
